# Supplementary material for: A Wipe-Based Stool Collection and Preservation Kit for Microbiome Community Profiling
Source: Front Immunol. 2022 May 30;13:889702. doi: 10.3389/fimmu.2022.889702 (PMC9196042; doi:10.3389/fimmu.2022.889702)
Supplement: Supplementary file 2 [file DataSheet_1.docx]

**Supplemental Materials**

**A wipe-based stool collection and preservation kit for microbiome community profiling**

Hui Hua, Cem Meydan, Evan E. Afshin, Loukia Lili, Christopher R. D’Adamo, Joel Dudley, Nathan D. Price, Nate Rickard, Bodi Zhang, Christopher E. Mason

**Supplemental Figures**

**
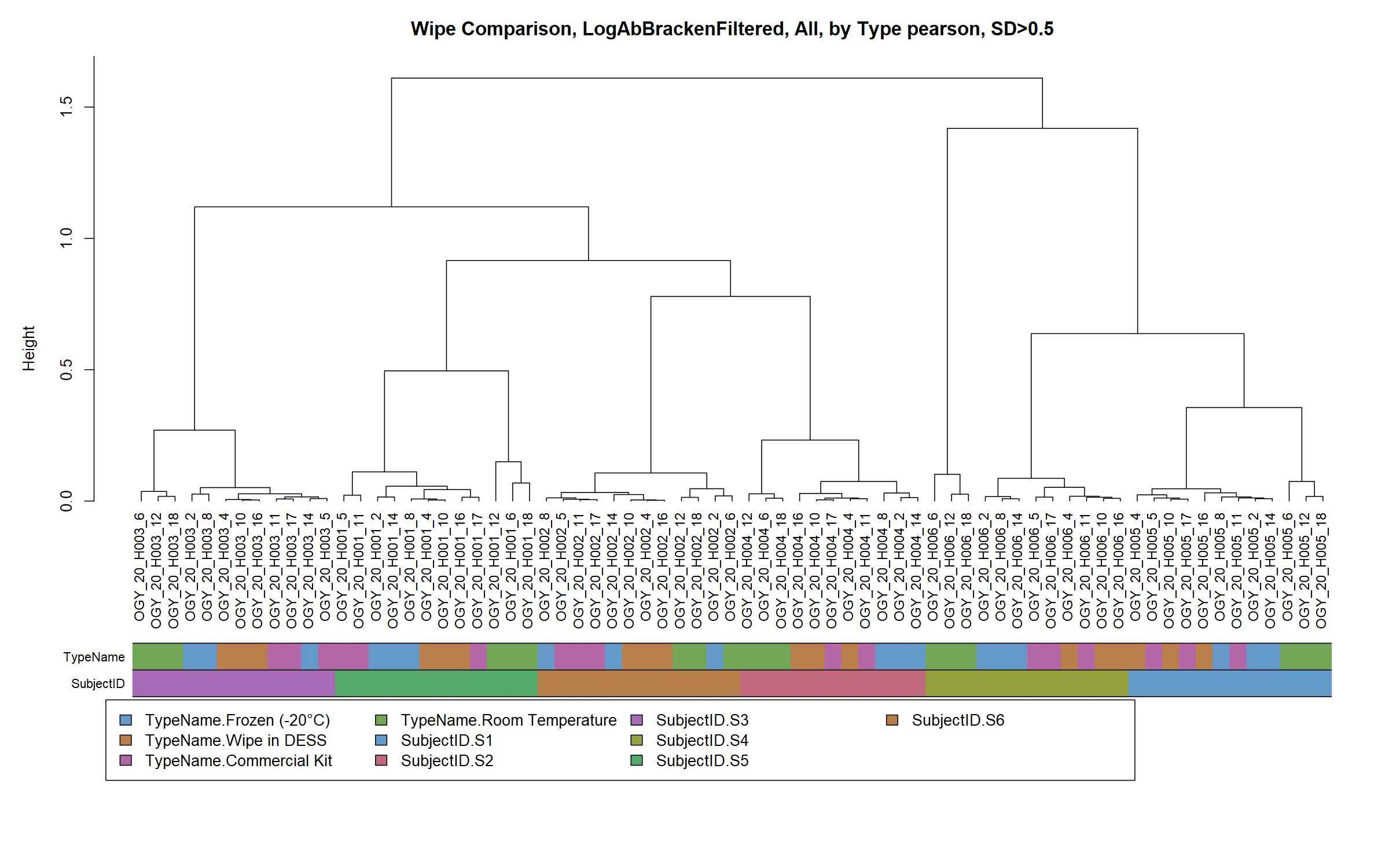
**

**Supplemental Figure 1. Sample Clustering Dendogram.** Dendrogram plot showing sample clustering and similarity by sample type and subject.

**Supplemental Figure 2. Sample Clustering Principle Coordinates Analysis.** Principle Coordinates Analysis (PCoA) plot that shows clustering by subject and sample type.

**
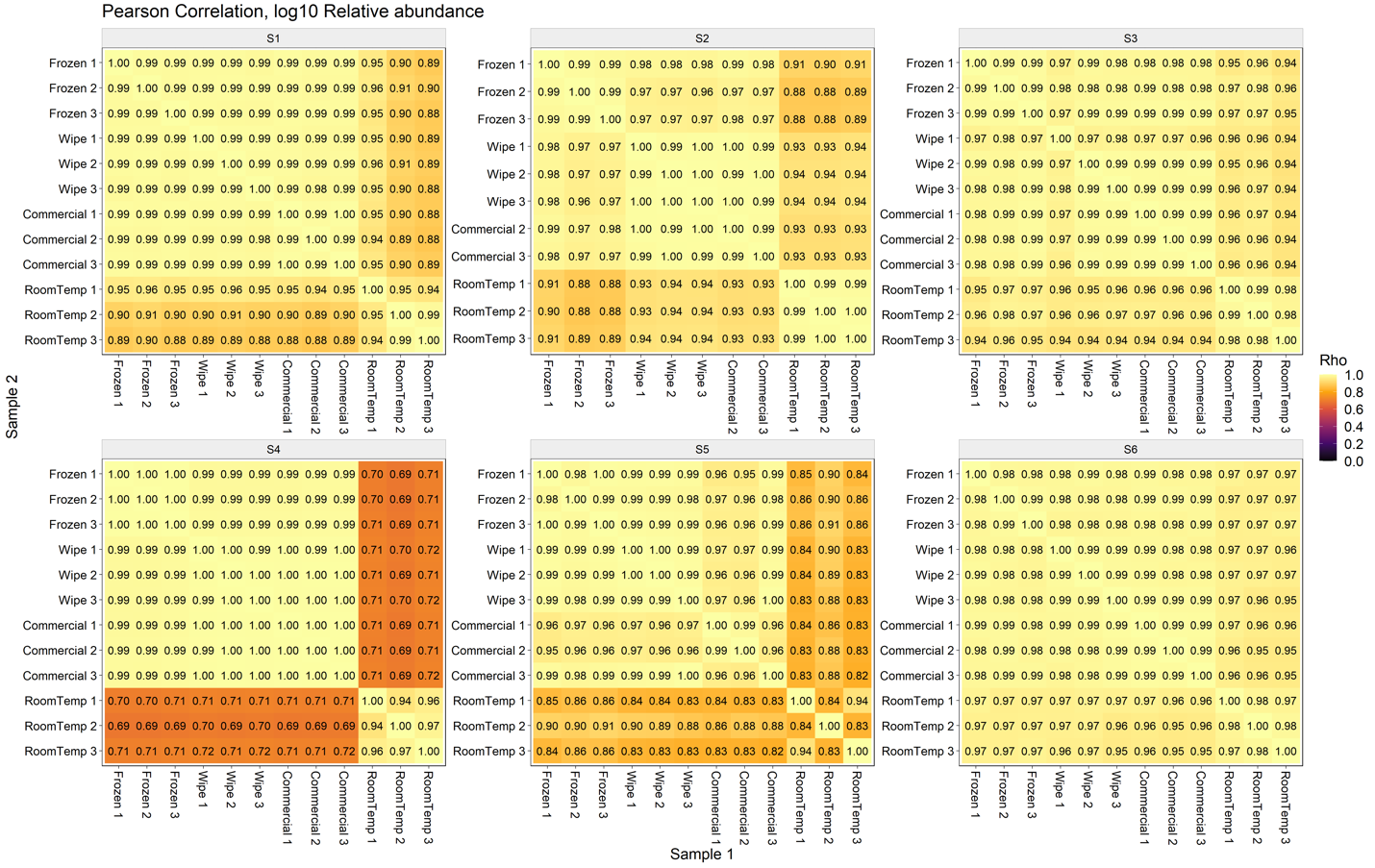
**

**Supplemental Figure 3. Taxonomic Pearson Correlation.** Pearson correlation of taxonomic relative abundances by sample type and subject.


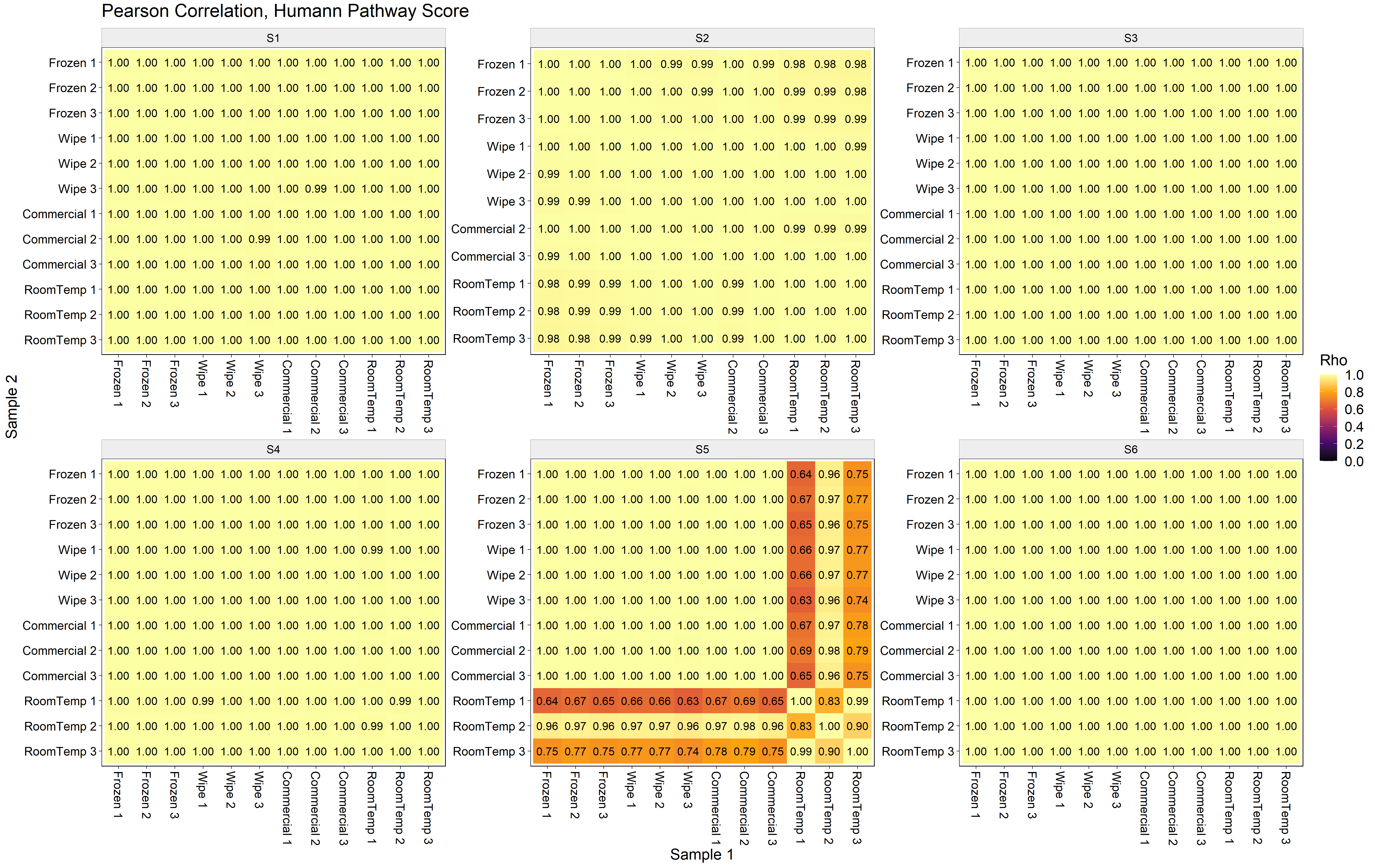

**Supplemental Figure 4. Functional Pathway Pearson Correlation.** Pearson correlation of HUMAnN pathway scores by sample type and subject.

**Supplementary Table 1. Demographic Data.** Summary demographic data from the six subjects.

**Supplementary Table 2. DNA Extraction Data.** Summary of DNA extraction yields from the different samples.

**Supplementary Table 3. Alpha Diversity Metric Statistics.** Summary of the means and standards of deviation of alpha diversity metrics.
